# New developments in the pathology of malignant lymphoma: a review of the literature published from May 2015–September 2015

**DOI:** 10.1007/s12308-015-0262-3

**Published:** 2015-11-19

**Authors:** J. Han van Krieken

**Affiliations:** Department of Pathology, Radboud University Medical Centre, P.O. Box 9101, 6500 HB Nijmegen, The Netherlands

**Keywords:** Pathology, Lymphoma

## Introduction

We are in the era of precision medicine, but I am not sure whether we are at the beginning, in the middle, or at the end. On the one hand, we have only a few targets in certain cancer types for which we have precise drugs, like the tyrosine kinase inhibition in chronic myeloid leukemia; on the other hand, it seems that targeting most cancers requires action towards several different pathways. This review indicates that our knowledge accumulates fast, but that simple solutions might not be expected anymore. Nevertheless, I do think that we are on the right path, but it will be a long one.

## Biology of lymphoma

### B cell lymphomas

A fine example of complexity but nevertheless potential new treatment options is given by the work of Thijssen et al. [1]. It is well known that activation of B cell receptor (BCR) signaling is important to keep chronic lymphocytic leukemia (CLL) cells viable. This pathway includes phosphoinositide 3-kinases (PI3K) and mTOR, but inhibition of PI3Kδ has little effect, both clinically and in vitro. Thijssen et al. used a pan-PI3k/mTOR inhibitor (voxtalisib), which induced apoptosis in CLL cells and in addition blocked adhesion and proliferation. Of course clinical studies are needed, since such a potent inhibition of critical pathways in many cells might not be applicable in patients due to side effects. Mantle cell lymphoma (MCL) is another B cell lymphoma in which BCR targeting has shown some improvement in outcome, but prolonged and/or complete responses are rare. Ahkter et al. [2] have performed detailed digital quantification of BCR signaling molecules and their effector pathways in tumor tissues from 81 MCL patients. They found two distinct (BCR^high^ and BCR^low^) subsets of patients: higher levels of BTK/SYK/BLNK/CARD11/PLCG and lower expression of MALT1/BCL10 genes was considered indicative of low BCR activation, amplified expression of TLR6/TLR7/TLR9 was regarded as BCR^high^; the latter group had also enhanced expression of genes associated with NF-κB pathway and a poorer clinical outcome. Also in follicular lymphoma (FL) BCR signaling is critical. In fact, there is aberrant glycosylation of the immunoglobulin receptor in FL as a result of which DC-SIGN can activate the lymphoma cells. Linley et al. [3] show that DC-SIGN induced binding, results in MYC activation and that no endocytosis of surface immunoglobulin occurs. The authors suggest that these findings indicate that, in contrast to CLL, specific antigen binding of the B cell receptor does not play a role in FL, but that monocytes that carry DC-SIGN drive the lymphoma cells.

Trying to understand the process of transformation of FL also does not result in an easy answer. Extensive work on many cases of transformed FL (>280 cases in total) by Kridel et al. [4] resulted in the finding that in most cases (about 80 %), transformed FL is of the germinal center B cell type (GBC), but that almost 20 % are of the activated B cell type (ABC), although, interestingly, this latter group is comprised mainly of bcl2 translocation negative cases (might these actually be transformed marginal zone lymphomas as suggested by van den Brand et al. [5–7]. IRF4 expression in FL indicates early (<5 years) transformation rather than later (but cases that had no transformation were not included in the study). Furthermore, cases that at transformation are still composite have a better prognosis.

Using large data sets in a clever way can provide insight in the complexity of lymphoma-immune response interaction and Care et al. [8] provide such an approach: using 10 publicly available gene expression data sets encompassing 2030 cases, they explored the nature of the host response in diffuse large B cell lymphoma (DLBCL). They show that T cell and cytotoxic gene expression with polarization along the IFNγ-STAT1-IRF1 axis is related to improved outcome, independent of the sub-classification into GBC and ABC types.

Burkitt lymphoma (BL) remains an intriguing lymphoma, for which we know already a lot of the molecular background and it is already well curable in children but not in adults. The genetic hallmark of BL is the translocation t(8;14)(q24;q32), or one of its light chain variants, resulting in IG-MYC juxtaposition, but other genetic alterations and Epstein-Barr virus (EBV) play a role as well. Piccaluga et al. [9] compared specific gene and microRNA expression in EBV-positive and EBV-negative BL. They found significant differences in the expression of viral microRNAs and in selected target genes: LIN28B, CGNL1, GCET2, MRAS, PLCD4, SEL1L, SXX1, and the tyrosine kinases encoding STK10/STK33. GCET2, also validated by immunohistochemistry, appeared to be a useful marker for distinguishing EBV-positive and EBV-negative cases. They conclude that their data indicate significant differences in the transcriptional profiles of EBV-positive and EBV-negative BL and highlight the role of virus-encoded miRNA. Wagener et al. [10]; the et al. being a huge group of collaborators (*n* > 150)) identified somatic mutations in PCBP1 in 3/17 (18 %) BL (interesting to see that such a large consortium describes so few cases…; and a few of the authors do have many more cases, see below). They confirmed the recurrence of PCBP1 mutations by Sanger sequencing in an independent validation cohort, finding mutations in 3/28 (11 %) BL (still not a lot of cases….) and in 6/16 (38 %) BL cell lines. In silico evaluation of the mutations indicates that these alter the function of the gene, which includes nuclear trafficking and pre-mRNA splicing. How this affects BL pathogenesis remains elusive. A subset of the collaborative group Doose et al. ([11]; still >100 researchers) studied the role of MYC in 2 B cell lines and 91 B cell lymphomas; they identified 13 long non-coding RNAs (lncRNAs) differentially expressed in MYC translocation-positive BL. They focused on an lncRNA that they named MYC-induced long non-coding RNA (MINCR) and that showed a strong correlation with MYC expression in MYC-positive lymphomas. MINCR knockdown appeared to be associated with impairment in cell cycle progression, suggesting that MINCR acts as a modulator of the MYC transcriptional program. They conclude by suggesting that MINCR is a newly identified player in the MYC transcriptional network able to control the expression of cell cycle genes. These three studies reveal once again that research is not driven by hypothesis results in lots of new data adding to complexity, but actually gives little real understanding or potential for translational research.

### T cell lymphoma

Hypothesis-driven research sometimes leads to unexpected findings. Jain et al. [12] set off to investigate the role of T cells in B cell lymphoma development. They investigated the spleens and lymph nodes from SJL/J mice that exhibit a high incidence of mature B cell lymphomas and found increased numbers of germinal centers and T follicular helper (T_FH_) cells. Microarray analyses revealed high levels of transcripts encoding IL-21 associated with high levels of serum IL-21. Then, they developed IL-21 receptor (IL21R)-deficient SJL mice, which appeared to have reduced numbers of T_FH_ cells, lower serum levels of IL-21, and few germinal center B cells, and did not develop B cell tumors. Surprisingly, they also noted features similar to human angioimmunoblastic T cell lymphoma (AITL), which is a malignancy of T_FH_ cells. Subsequently, they performed gene expression analyses of human AITL samples and showed that all cases expressed elevated levels of transcripts for IL21, IL21R, and a series of genes associated with T_FH_ cell development and function. So, unexpectedly, they have developed a mouse model with features of AITL based on which they suggest that patients with this disease might benefit from therapeutic interventions that interrupt IL-21 signaling.

Anaplastic lymphoma kinase (ALK)-positive anaplastic large cell lymphoma (ALCL) is unusual among the lymphoma types, since it has a clear driver mechanism that it shares with other malignancies, especially ALK-positive lung cancer. Therefore, targeted treatments are studied across cell of origin concepts, but there appear to be still cell type-dependent resistance mechanisms. Crizotinib is the best known ALK tyrosine kinase inhibitor and is used to treat ALK-associated cancers. Mitou et al. [13] showed that autophagy is activated in ALCL cells submitted to ALK inactivation as a resistance mechanism. Furthermore, they show that co-treatment with crizotinib and chloroquine prevents autophagy as escape mechanism and thus could be beneficial for ALK-positive ALCL patients.

## Epidemiology of lymphoma

Cancer incidence changes, due to different reasons, one of them being changes in diagnostic criteria. To understand whether Hodgkin lymphoma (HL) really is changing in appearance or that diagnostic criteria give that impression, Glaser et al. [14] analyzed detailed histology-specific classical (c)HL incidence rates in 1992 through 2011 US SEER data (*n* = 21,372) and reviewed pathology reports from a regional subset of 2007 through 2011. cHL rates were stable until 2007 and then decreased. Nodular sclerosis rates declined after 2007 by 6 % annually, with variation by gender, age, and race/ethnicity. In 1992 through 2011, mixed cellularity rates declined, whereas not otherwise specified (NOS) rates rose. Eighty-eight of 165 reviewed NOS pathology reports addressed classification choice. Twenty (12 %) justified the classification, 21 (13 %) described insufficient biopsy material, and specific subtype information was missing for 27 (16 %). They conclude that recent nodular sclerosis rate declines largely represent true incidence changes but that rate decreases for mixed cellularity and other less common subtypes, and increases for NOS (comprising ∼30 % of cHL cases in 2011!!), likely reflect changes in diagnostic and/or classification practice.

One of the most serious consequences of organ transplantation is the occurrence of post-transplant lymphoproliferative disease (PTLD), especially problematic in heart and lung transplantation. Kuramarasinge et al. [15] describe 70 PTLD from their institution (41 heart, 22 lung, 6 heart-lung, and 1 heart-kidney transplant) from 1984 to 2013. The incidence of PTLD was almost 8 % in heart-lung, 5 % in heart, and 3 % in lung transplant recipients. Extranodal disease (82 %) with DLBCL (72 %) was the most common presentation. Bone marrow involvement (13 %) and central nervous system disease (3 %) were uncommon. Heart transplant recipients had later onset of PTLD (>1 year post-transplant), with less allograft involvement, compared with lung and heart-lung recipients. Poor prognostic markers were bone marrow involvement and serum albumin. Improved survival was seen with a complete response within 3 months of treatment. A 5-year overall survival was 29 %. Of course, with data collected over such a long time, changes in early detection (monitoring EBV levels in the blood) and treatment (introduction of rituximab) cannot be evaluated with enough detail, but the disease remains an important problem in transplantation.

## Defining entities

### B cell lymphomas

Lymphomas are classified based on morphology, phenotype, genotype, and clinical features, and mediastinal (m)DLBCL is one of the entities in which localisation is key for its recognition (in contrast to for instance the nasal NK/T cell lymphoma, which we refer to as nasal NK/T-cell type in case it presents in the skin or intestine (see below) or the leg-type DLBCL that can present anywhere in the skin). It therefore does make sense to investigate whether mDLBCL can present at other sites, where it should then be diagnosed as mDLBCL type. Yuan et al. [16] used their DLBCL gene expression profile to see whether this can be the same in DLBCL that present outside the mediastinum. Underlying this approach is the assumption that actually lymphoma classification according the 4 mentioned criteria is not the best approach, but that gene expression profiling is the real gold standard (see below!). In their series of DLBCL cases, they identified 24 cases with a signature of mDLBCL, including 9 cases (that all had mediastinal involvement at presentation) with a submission diagnosis of DLBCL consistent with mDLBCL (G-DLBL-P) and 15 cases with a submission diagnosis of DLBCL (9 of which had mediastinal involvement!). The cases were reviewed and the reviewers agreed with the diagnosis in the 9 G-mDLBCL-P cases. Among the other 15 DLBCL cases, 11 were considered to be mDLBCL or DLBCL consistent with mDLBCL (a very subtle difference it seems to me), 3 were considered to be DLBCL, and 1 case was a gray-zone lymphoma with features intermediate between DLBCL and cHL. Only 6 of the cases had no clinical or radiologic evidence of mediastinal involvement. Surprisingly, the authors conclude that mDLBCL can present as a non-mediastinal tumor without evidence of mediastinal involvement and that gene expression profiling offers a more precise diagnosis of mDLBCL. In fact, the data indicate that gene expression profiling lumps different well-characterized clinicopathologic entities into one group: a fine example of not trying to reject your hypothesis, but to fit your data into your hypothesis.

Lu et al. [17] as well as Ok et al. [18] have a more open-minded approach: they investigate whether in EBV-positive DLBCL age is relevant. As I wrote previously in one of the literature reviews, the age cutoff for EBV-positive DLBCL of the elderly may be associated with the age of the diagnosing pathologist and is not settled. By looking at all EBV-positive DLBCL in their patients, regardless of age, Lu et al. [17] found that patients with EBV-positive DLBCL shared many unfavorable prognostic characteristics, regardless of age. EBV-positive cases, both in the elderly and young groups, showed significantly worse overall survival and progression-free survival than negative cases. Moreover, no significant difference of outcome was identified between different age groups with EBV-positive DLBCL. In conclusion, EBV-positive DLBCL patients, regardless of age, shared similar poor prognostic features and showed worse outcome than negative cases. They suggest that the age criterion of EBV-positive DLBCL of the elderly, and possibly the name itself, should be modified in future. The same conclusion is drawn by Ok et al. [18] who evaluated the clinicopathologic, immunophenotypic, and genetic features in Caucasian patients with EBV-positive DLBCL who are ≤50 years of age and compared this patient group to patients who are >50 years. In patients who are ≤50 years, less frequent expression of BCL6 and a trend of more frequent expression of CD30 and pSTAT3 were found in patients with EBV-positive DLBCL. In patients who are >50 years, common expression of CD30, p50, pSTAT3, and less frequent expression of BCL6 were observed. Older patients also more commonly had a poor performance status. Both groups had similar clinicopathologic, immunophenotypic, and genetic features. Gene expression profiling, microRNA profiling, and treatment outcome of the younger patients with EBV-positive DLBCL was not distinctive from tumors in older patients. Based on the data from both groups I believe there is sufficient evidence that the arbitrary age cutoff for EBV-positive DLBCL should be eliminated in the WHO lymphoma classification.

Pathologists like to see what happens in a tumor rather that relying on something that comes out of a sequencing machine or is seen in a dark room with fluorescence and actually that makes some sense: the results of genetic changes are what counts for a cell. Agarwal et al. [19] studied 209 lymphoma cases (15 BL, 13 intermediate BL/DLBCL, and 181 DLBCL) using immunohistochemistry and fluorescent in situ hybridization (FISH) for MYC staining and rearrangement. They showed that using a cutoff value of 30 % indicated not only a high chance that there is a MYC rearrangement but also conclude that interlaboratory validation is needed as well as FISH confirmation.

### T cell lymphomas

As discussed above, extranodal NK/T cell lymphoma, nasal type, may present outside of the nasal region. Fang et al. [20] describe 10 cases that presented in the intestine: 1 patient had stage I disease, 7 had stage II disease, and 2 had stage III disease; 8 of the 10 patients had lymphadenopathy and no patient had bone marrow involvement; all patients had a low International Prognostic Index (IPI) score (<3) at presentation; the median age at the time of diagnosis was 37.5 years (range = 24–68 years); all the patients died within 2 years with a median survival time of less than 10 months. Except for the size of tumor cells, most of the morphologic and immunophenotypical features of the cases were similar to those involving nasal region. These data indicate that indeed these cases belong to the same entity.

### Cutaneous lymphomas

Recognizing the leg-type DLBCL of the skin requires a set of antibodies but remains sometimes problematic. Robson et al. [21] investigated whether p63 expression might be helpful: 21/30 of their cases of leg-type DLBCL but only 4/34 cases follicle center cell lymphoma were staining positive for p63; this expression correlated strongly with proliferation rate as assessed by Ki-67. Although the mechanism remains unclear, this may be of additional diagnostic value.

## New entities/subtypes

Tumors like to confuse pathologists, especially those who rely very heavily on immunohistochemistry to classify lymphomas. Experienced pathologists of course know that lymphomas do not read our books and cancer cells do have aberrations, including aberrant phenotypes. It is therefore of no surprise that CD10-positive MCL exists and Akhter et al. [22] wondered whether this aberrant expression was clinically relevant. Their series of 9 CD10-positive MCL cases showed a distinct RNA expression profile of a germinal center signature but with minimal impact on downstream signaling pathways. This is an interesting finding, indicating that one has to take great care to use gene expression profiling as the gold standard for classification (see above!) There were no significant differences in the clinicopathological features including outcome between the CD10-positive and CD10-negative MCL cases. Although the numbers are small, the authors conclude that their study provides convincing evidence that CD10 expression is related to a distinct gene expression signature in MCL, but without clinical or biological implications. These cases therefore remain bona fide MCL cases, which supports my hypothesis that gene expression profiling is of less relevance than protein expression; of course, I realize that more cases are needed to reject my hypothesis….

Another example of an aberrant feature in MCL is plasma cell differentiation. Ribera-Cortada et al. [23] investigated the terminal B cell differentiation phenotype in 60 MCL, because in vitro work had shown that SOX11, which is commonly expressed in MCL, suppresses a plasma cell differentiation program. Indeed, monotypic plasma cells and lymphoid cells with plasmacytic differentiation (and expressing cyclin D1) were observed in 7/20 (37 %) SOX11-negative, but in none of the 41 SOX11-positive MCL. Furthermore, BLIMP1 and XBP1 expression, indicative of plasma cell differentiation, was also more frequent in SOX11-negative than in SOX11-positive cases (83 vs. 34 and 75 vs. 11 %, respectively). However, no difference in the expression of IRF4/MUM1 was observed. The authors conclude that their results indicate that SOX11-negative MCL may be a particular subtype of this tumor characterized by more frequent morphological and immunophenotypic terminal B cell differentiation features that may be facilitated by the absence of SOX11 transcription factor; however, more molecular and clinical data are needed to reject the hypothesis that the findings are just a minor aberration from the general, something very common in all kinds of cancer.

Magro et al. [24] did a large effort to collect cases of the extremely rare epidermotropic marginal zone B cell lymphoma (eMZL). They had 2 own cases and tried to collect tissue and data from the 5 cases reported in the literature (from 3/5 this was received). All patients were elderly, 6 males, 1 female, and 6/7 patients presented (the female was the exception) with a pityriasis rosea-like presentation. Despite bone marrow, blood, and/or spleen involvement at presentation or within months, none of the patients died of disease. The biopsies of the 5 cases showed areas of superficial band-like lymphocytic infiltration with large monocytoid appearance and an epidermotropic pattern of lymphocyte migration into the epidermis. Neoplastic cells were extensively positive for CD20, CD79a, and BCL-2 and negative for CD10 and BCL-6. There was also expression of CRCX3. Although this process seems to be very rare, the data indicate that this may represent indeed a separate entity; more cases need to be collected however.

Deng et al. [25] go for a more complete approach to show that there is a relevant subtype of DLBCL. They studied a cohort of 587 patients with DLBCL for hepatitis virus B (HBV) infection status, clinicopathologic features, and the immunoglobulin variable region. Eighty-one (14 %) of their patients were HBV positive. This is likely larger than can be expected in a western population since HBV infection is more common in China. These patients presented at younger age than HBV-negative patients (45 vs. 55 years), had more frequent involvement of the spleen or retroperitoneal lymph nodes (41 vs. 16 and 62 vs. 31 %, respectively), more advanced disease (stage III/IV 77 vs. 60 %), and a worse outcome (2-year overall survival 47 versus 70 %). In HBV-positive DLBCL patients, almost all (45/47, 96 %) amino acid sequences of heavy and light chain complementarity determining region 3 exhibited a high homology to antibodies specific for HBV surface antigen, and the majority (45/50, 90 %) of IgHV and IgLV genes were mutated. The authors hereby show compelling evidence that these cases indeed comprise a separate subset of DLBCL. It would be quite interesting to investigate how often such cases occur in other regions in the world and whether treatment of the HBV could affect the course of the lymphoma.

The diagnosis of BL can be challenging in adults, especially in case there is expression of BCL2. A few authors from the group that studied BL for gene and MYC expression in a relatively small series of cases (see above) present the expression of BCL2 in 150 cases of conventionally diagnosed BL using two different BCL2 antibodies [26]. BCL2 expression was detected in 23 % (!!) of the cases, though the expression varied in intensity and number of positive cells. There was no relevant difference in clinical presentation and outcome between BCL2-positive and BCL2-negative BL in a subgroup of 43 cases for which detailed clinical data were available. An independent cohort of 17 BL with expression of BCL2 were analyzed molecularly, with 13 of 17 cases classified as molecularly defined BL using gene expression profiling on formalin-fixed paraffin-embedded tissues. This indicates that BCL2 expression in BL is another example of lymphomas not doing what we like them to do. Actually, I know that some (many?) pathologists reject a diagnosis of BL in case BCL2 is negative.

Classification of T cell lymphomas is difficult, but the AILD-type has relatively well defined criteria. Wang et al. [27] complicate the situation by describing a subgroup defined by IDH2R172 mutations. They performed targeted sequencing on 92 cases of peripheral T cell lymphomas (PTCL) and identified frequent mutations affecting RHOA, TET2, DNMT3A, and isocitrate dehydrogenase 2 (IDH2). Although IDH2 mutations were largely confined to AITL, mutations of the other 3 were found in other types of PTCL as well. AITL cases with IDH2(R172) mutations demonstrated a distinct gene expression signature characterized by downregulation of genes associated with T_FH_ differentiation (e.g., STAT1 and IFNG) and enrichment of an interleukin 12-induced gene signature. Tissue samples with IDH2 mutations displayed a prominent increase in H3K27me3 and DNA hypermethylation of gene promoters. However, data regarding clinical features were not presented, so it is not clear whether this is just a phenomenon of variation within an entity or a real subgroup.

With the increasing availability of whole genome sequencing increasingly also rarer tumor types are completely sequenced. Ungewickell et al. [28] report recurrent point mutations and genomic gains of TNFRSF1B, encoding the tumor necrosis factor receptor TNFR2, in 18 % of patients with mycosis fungoides (MF) and Sézary syndrome. In vitro expression of the recurrent TNFR2 Thr377Ile mutant in T cells leads to enhanced non-canonical NF-κB signaling that is sensitive to the proteasome inhibitor bortezomib. Using an integrative genomic approach, we additionally discovered a recurrent CTLA4-CD28 fusion, as well as mutations in downstream signaling mediators of these receptors. These findings may indicate potential new treatment options.

## Pitfalls in lymphoma diagnosis

Diagnosing lymphomas after treatment can be quite difficult, but sometimes the clinical situation is such that a biopsy can only be taken after some treatment. This may especially be the case in primary central nervous system lymphomas (PCNSL). Onder et al. [29] analyzed the cytological, histopathological, and immunohistochemical features in stereotactic biopsies of 25 primary CNS lymphoma cases pre-treated with corticosteroids. In 48 % of the cases, the diagnosis was straightforward. These cases were characterized by prominent centroblasts either in diffuse parenchymal infiltrates or in perivascular regions. The remaining 52 % demonstrated some degree of variability in diagnosis among pathologists. Lymphoid atypia (other than the typical blastic morphology) appeared as a subjective finding and this was more pronounced in cytology preparations. In our study, corticosteroid pre-treatment in primary CNS lymphoma was associated with a large spectrum of histopathological, immunohistochemical, and cytological findings. Even the combined use of an extended immunohistochemical panel left the diagnosis open to subjectivity.

## Prognostic factors in lymphoma

In my previous review of the literature [30], I was positive regarding the move from prognostic to predictive factors, but maybe I was overoptimistic. In this review, there are again quite some studies, but mainly pure prognostic, and therefore likely of little clinical relevance.

Cencini et al. [31] investigated whether there is a correlation between 2 factors that indicate relapse in HL: tissue macrophage infiltration and early PET-positivity after treatment. In a cohort of 200 HL patients early FDG-PET was positive in 37 patients (19 %). CD68 expression was low, intermediate, or high in 26 (13 %), 100 (50 %), and 74 (37 %) cases, without difference in the distribution between responders and non-responders and the score did not show any correlation with early FDG-PET result. These authors therefore rejected their hypothesis that the level of macrophage infiltration correlates with FDG-PET assessment.

Suppressor of cytokine signaling 1 (SOCS1) mutations are among the most frequent somatic mutations in HL, yet their prognostic relevance is unexplored; for Lennerz et al. [32] sufficient reason to do so. In 105 HL samples, they found a mutation in SOCS1 in about 60 %. They found no clinicopathologic differences between the patients with mutated HL versus those without; however, patients with major SOCS1 mutations (i.e., mutation that decrease the length of the protein in contrast with those that have non-truncating mutations) had early relapse and shorter overall survival.

Pastore et al. [33] performed targeted deep sequencing for 74 genes in 151 FL biopsy specimens that were obtained from patients treated with rituximab, cyclophosphamide, doxorubicin, vincristine, and prednisone (R-CHOP). Mutations and clinical factors were incorporated into a risk model for failure-free survival, and this was tested in an independent population-based cohort of 107 patients. The risk model, which they termed m7-FLIPI, included the mutation status of seven genes (EZH2, ARID1A, MEF2B, EP300, FOXO1, CREBBP, and CARD11), the follicular lymphoma (FL)IPI, and Eastern Cooperative Oncology Group (ECOG) performance status. In both the training and validation cohort, m7-FLIPI defined a high-risk group of about 25 % with a 5-year failure-free survival of 30 versus 70 % for the low-risk group which outperformed a prognostic model of only gene mutations, FLIPI alone, and FLIPI combined with ECOG performance status. However, the impact of adding mutational data to FLIPI plus ECOG is not very large, so it is questionable whether m7-FLIPI will enter the clinic.

The work of Novak et al. [34] brings new developments together, resulting in new data: By doing whole genome sequencing, they find novel genomic alterations in DLBCL and by selecting patients with modern treatment, they target the right patient population. Doing this on only 51 patients is understandable, but also precludes definitive conclusions. They identified 16 genes with mutations, 374 with copy number gains and 151 with copy number losses that were associated event-free survival. One gene (SLC22A16) at 6q21, a doxorubicin transporter, was lost in 54 % of DLBCL of patients with poor outcome. Such a functional explanation of prognostic indicator makes it potentially a predictive marker and thus potentially of real clinical value.

Also, exome sequencing is a powerful tool, and Jiang et al. [35] hereby identified somatic gene mutations in 25 patients with NK/T cell lymphoma and confirmed them in an extended validation group of 80 people by targeted sequencing. Recurrent mutations were most frequently located in the RNA helicase gene DDX3X, tumor suppressors (TP53 and MGA), JAK-STAT-pathway molecules (STAT3 and STAT5B), and epigenetic modifiers (MLL2, ARID1A, EP300, and ASXL3). Clinically, patients with DDX3X mutations presented a poor prognosis. Further work is needed to see how this may result in altered treatments for this tumor with a generally quite poor prognosis.

Okamata et al. [36] investigated the prognostic value of EBV in biopsies and in blood from patients with DLBCL. Among 140 DLBCL patients without underlying immunodeficiency, 51 were evaluable for both EBER and EBV DNA load, 83 for EBER only, and one for EBV DNA load only. Importantly, EBV DNA load was higher for EBER-positive patients but EBV DNA was detected also in up to 72 % of EBER-negative patients. Progression-free survival and overall survival were significantly worse for patients with EBV DNA load above 700 copies/μg, and for EBER-positive patients and even among EBER-negative patients, higher EBV DNA load conferred worse progression-free survival and overall survival. These findings indicate that EBV DNA load in diagnostic specimens is not a simple surrogate for the EBER status of the lymphoma and may be a potential biomarker associated with EBV involvement and prognosis in DLBCL. The same group also published this approach using data from 127 patients with similar results [37]. In 15 patients (12 %), Epstein-Barr virus DNA was detected and these patients were older and tended to be at a more advanced disease stage and had a poorer outcome. The EBER status was known for 123 patients; 6 of 8 positive patients (75 %) and 9 of 115 negative patients (8 %) had detectable EBV DNA in pre-treatment serum.

Huntingtin-interacting protein 1-related (HIP1R) is an endocytic protein involved in receptor trafficking, including regulating cell surface expression of receptor tyrosine kinases. Wong et al. [38] had shown that low HIP1R protein expression was associated with poorer survival in DLBCL patients treated with R-CHOP and now extend these findings. Thanks to the movement of open access of data, they were able to use data mining on three independent transcriptomic datasets of DLBCL. The HIP1R transcript was more often expressed in GCB-like DLBCL subtype and lower expression was correlated with worse overall and progression-free survival. Using immunohistochemistry, HIP1R expression at 30 % cutoff was associated with GCB-DLBCL molecular subtype and predictive of survival in DLBCL patients treated with R-CHOP (*n* = 73). Cases with high FOXP1 and low HIP1R expression frequency exhibited poorer survival. They conclude that HIP1R expression is strongly indicative of survival when utilized on its own or in combination with FOXP1, and the molecule is potentially applicable for subtyping of DLBCL cases. This latter conclusion goes a bit far, since the expression of these markers is not a better classifier that the previously published classifiers.

Wang et al. [39] evaluated the expression profiles and the clinical significance of BAFF and BAFF-R DLBCL in paraffin-embedded specimens from 136 patients with newly diagnosed DLBCL treated with R-CHOP. BAFF and BAFF-R were expressed in 72 % (98/136) and 47 % (64/136), respectively, and negative BAFF-R but not BAFF expression was correlated with poor prognostic factors like, elevated serum lactate dehydrogenase levels, a high (R-) International Prognostic Index score, a lower complete response rate, and inferior survival. Liu et al. [40] investigated survivin expression in 463 R-CHOP-treated DLBCL patients: 269 (58 %) had survivin overexpression (cutoff of >25 % positive cells), associated with high IPI score, disease in ≥2 extranodal sites, and a high Ki-67 index; in ABC type, survival of survivin-positive patients was lower; cases with wild-type p53 and surviving overexpression had lower survival rates; in STAT3-positive cases, survivin overexpression was associated with better survival. Kiyashu et al. [41] stained 1253 DLBCL samples with programmed cell death ligand 1 (PD-L1) and established a new definition of PD-L1-positive DLBCL and microenvironment positive cases (mPD-L1+). Of 273 patients, clinical information was available. There were 11 % PD-L1-positive and 15 % mPD-L1-positive cases, both associated with the ABC type and EBV positivity. Patients with PD-L1-positive DLBCL had inferior overall survival, but there was no difference in survival between mPD-L1-positive and mPD-L1-negative DLBCL. The authors suggest that immunotherapy targeting the PD-1/PD-L1 pathway should be considered in this distinct DLBCL subgroup, but have no experience with such an approach. Wang et al. [42] show that in 98 patients with DLBCL, the clinicopathologic features were very similar between the CD30-positive and CD30-negative groups. The only major difference was that CD30 expression was nearly exclusively seen in cases without MYC rearrangement. CD30 expression was not predictive of overall survival irrespective of therapy regimens, ABC vs. GBC type.

## Staging

Several techniques can detect very low levels of tumor cells (minimal residual disease (MRD). Drandi et al. [43] used Droplet Digital PCR in comparison with a standard qPCR approach to detect rearranged immunoglobulin genes, as determined on the primary lymphoma, in the blood of patients with multiple myeloma, MCL, and FL (overall 222 samples). There was a good concordance of both methods with 189 of 222 samples being fully concordant. Therefore, they conclude that ddPCR is a reliable tool for MRD detection, which has a greater applicability and reduced labor intensiveness than qPCR.

Hurabielle et al. [44] investigated the value of the detection of MRD in MF after 1 year of treatment, which had been shown of prognostic relevance in retrospective studies. They performed a prospective study and at diagnosis, 126 of 204 cases (62 %) showed a T cell receptor clone in the skin lesion. After 1 year, 83 of 178 patients (47 %) still being followed up were in CR and 13 of 63 (21 %) showed MRD. The presence of MRD did not predict clinical status at 4 years. This large amount of work shows how important it is to confirm the value of biomarkers in prospective studies.

## Ancilliary techniques

Deep learning, an approach combing Big Data and novel bioinformatics tools (3 buzz words in only 10 words!), is increasingly popular: it uses large amounts of data to create new knowledge without the bias of a hypothesis (at least that is the theory). Deeb (nomen est omen) et al. [45] present a state of the art quantitative mass spectrometric pipeline to characterize formalin-fixed paraffin-embedded (FFPE) tissues of patients with closely related subtypes of diffuse large B cell lymphoma (DLBCL). They quantified almost 9000 tumor proteins in 20 patients (!). This approach allowed the segregation of DLBCL patients according to ABC and GBC type. Then they extracted candidate proteins with the highest segregating power resulting in a panel of four proteins (PALD1, MME, TNFAIP8, and TBC1D4). However, confirmation on much larger case series is obviously needed.

Insuasti-Beltran et al. [46] developed a new pyrosequencing assay for MYD88 mutation detection. The MYD88 L265P mutation was identified in 43 cases of LPL (96 %), in only 2 cases of MZL (4 %), and 5 cases of CLL (2 %), thus confirming the strong association of the MYD88 L265P mutation with LPL, as well as the existence of rare cases of small B cell lymphoma that complicate this association.

Ryan et al. [47] used a novel approach called “pinpointing enhancer-associated rearrangements by chromatin immunoprecipitation,” or PEAR-ChIP, to simultaneously map enhancer activity and proximal rearrangements in lymphoma cell lines and patient biopsies. This method detects rearrangements involving known cancer genes, including CCND1, BCL2, MYC, PDCD1LG2, NOTCH1, CIITA, and SGK1, as well as novel enhancer duplication events of likely oncogenic significance. They found lymphoma subtype-specific enhancers in the MYC locus that are silenced in lymphomas with MYC-activating rearrangements and are associated with germline polymorphisms that alter lymphoma risk. BCL6-locus enhancers are acetylated by the BCL6-activating transcription factor MEF2B and can undergo genomic duplication or target the MYC promoter for activation in the context of a “pseudo-double-hit” t(3;8)(q27;q24) rearrangement linking the BCL6 and MYC loci. This highly interesting approach provides real new insights regarding enhancer-driven oncogene activation in lymphoma.

Sha et al. [48] use a bioinformatics approach on published data sets. They compared two published classifiers that distinguish BL from DLBL, something I did with lots of efforts by Southern blotting long time ago [49, 50]. Based on their analysis, they developed a new BL/DLBCL classifier and tested it on their own paraffin-embedded samples, allowing comparison with the diagnosis made in a central haematopathology laboratory and evaluation of clinical relevance. They show that both previous classifiers can be recapitulated using very much smaller gene sets than originally employed and exhibits high agreement (∼95 %) with the original diagnosis. Patients with lymphomas with intermediate features on conventional criteria were tested, and the cases classified as BL by the new classifier have worse response to standard DLBCL treatment than those classified as DLBCL. The classifier is available as a free software package through the link http://www.bioinformatics.leeds.ac.uk/labpages/softwares/ or on github https://github.com/Sharlene/BDC.

All and this selection from the vast literature hopefully provides you with some new insights and some helpful new tools but probably also with the realization that the world of lymphomas is not loosing its complexity.
